# The effect of time to surgery on clinical outcomes and hospitalization costs in older adults with femoral shaft fractures: A nationwide retrospective cohort study in Japan

**DOI:** 10.1007/s00068-026-03106-7

**Published:** 2026-02-23

**Authors:** Gaku Gondo, Daisuke Takada, Susumu Kunisawa, Kiyohide Fushimi, Yuichi Imanaka

**Affiliations:** 1 Department of Healthcare Economics and Quality Management, School of Public Health, Graduate School of Medicine, Kyoto University, Kyoto, Japan; 2 Department of Food Science and Nutrition, Faculty of Human Life and Science, Doshisha Women’s college of Liberal Arts, Kyoto, Japan; 3 Department of Health Policy and Informatics, Institute of Science Tokyo Graduate School of Medical and Dental Sciences, Tokyo, Japan; 4 Department of Health Security System, Centre for Health Security Graduate School of Medicine, Kyoto University, Kyoto, Japan

**Keywords:** Femoral shaft fracture, Mortality, Time to surgery, Early surgery, Elderly patients

## Abstract

**Purpose:**

This study aimed to evaluate the effect of early surgery on clinical outcomes and hospitalization costs in older adults with femoral shaft fractures.

**Methods:**

We conducted a retrospective cohort study using a nationwide administrative database in Japan, including patients aged 65 years and over who underwent surgery for femoral shaft fractures between 2014 and 2023. Early surgery was defined as surgery performed on the day of admission or the day after admission. We applied inverse probability of treatment weighting (IPTW) using propensity scores to adjust for confounding. Multiple imputation by chained equations was performed to impute missing data. The primary outcome was in-hospital mortality; secondary outcomes included complications, length of postoperative hospital stay, and hospitalization costs.

**Results:**

Among 11,087 eligible patients, 3,170 (28.6%) received early surgery. After IPTW adjustment, there was no significant difference in in-hospital mortality (odds ratio (OR), 0.96; 95% confidence interval (CI), 0.66–1.41). However, early surgery was associated with significantly lower odds of pneumonia (OR, 0.64; 95% CI, 0.44–0.94) and urinary tract infection (OR, 0.66; 95% CI, 0.48–0.90), shorter postoperative hospital stay (mean difference, 5.37 days; 95% CI, 3.56–7.19), and reduced hospitalization costs (mean difference, 150,380 Japanese yen; 95% CI, 93,270–207,490).

**Conclusion:**

Early surgery for femoral shaft fractures in older adults was not associated with a significant reduction in in-hospital mortality compared to delayed surgery. However, it was associated with fewer complications, shorter postoperative hospital stays, and reduced hospitalization costs. These findings suggest that early surgery may be beneficial for improving postoperative recovery.

**Supplementary Information:**

The online version contains supplementary material available at 10.1007/s00068-026-03106-7.

## Introduction

Femoral shaft fractures exhibit a bimodal age distribution, predominantly occurring both in young people and older adults. In youths, femoral shaft fractures are often caused by high-energy trauma, such as that experienced in motor vehicle collisions [1]. In older adults—where there are 40.8 cases per 100,000 people—many fragility fractures or atypical femoral fractures are associated with long-term bisphosphonate use [2–5].

Time to surgery is a modifiable factor that affects surgical outcomes for femoral fractures. Early surgery is effective in cases of severe or high-energy trauma, which frequently occur in younger individuals [6–10]. Many countries’ guidelines also recommend early surgery for hip fractures in older adults [11–13]. In Japan, reimbursement for surgery within 48 h of injury has been introduced for older adults with hip fractures, and early surgery for distal femur fractures has similar benefits [14]. Therefore, evidence already exists supporting early surgery for femoral fractures other than shaft fractures in older adults. However, few studies have evaluated the efficacy of early surgery for older adults with femoral shaft fractures, excluding severe or high-energy trauma.

In this study, patients with severe or high-energy trauma were excluded based on ICD-10 codes or early admission to the intensive care unit (ICU) or emergency ward to focus on low-energy trauma in older adults. This study aimed to examine treatment patterns and the effect of time to surgery on clinical outcomes for femoral shaft fractures in older adults, excluding cases involving high-energy or severe trauma, using a nationwide administrative claims database in Japan.

## Materials and methods

### Data source

This study was a multicenter retrospective cohort study that used the Diagnosis Procedure Combination (DPC) database of the DPC research group, which is funded by the Ministry of Health, Labour and Welfare in Japan. This database contains anonymized data voluntarily provided from over 1,000 hospitals and includes data from approximately 7 million patients per year, accounting for more than 50% of all acute care inpatients in Japan [15, 16]. The DPC database contains medical claim data, including hospital codes, clinical information and outcomes of inpatients, and information on medical procedures and fees [17]. These data are widely used in studies aimed at improving clinical practice, hospital management, and healthcare policy.

### Study cohort

Patients aged 65 years and over who were admitted and discharged between April 2014 and March 2023 and underwent surgery for femoral shaft fractures were included in this study. This age cutoff was adopted in accordance with the official definition of ‘older adults’ used in public materials in Japan [18]. Femoral shaft fractures were defined by ICD-10 code S72.30, and surgical procedures were defined using Japanese procedure billing codes, including open reduction and internal fixation of the femur (K0461). The following cases were excluded from this study: patients with an admission route other than home or facility (e.g., transfers from other hospitals or unknown routes) to ensure a clear definition of the ‘pre-admission living status’ covariate, which was a necessary component of the analysis; planned admissions because the condition and treatment procedures differ from those of emergency surgery cases; diagnoses containing the terms “postoperative,” “suspected,” or “implant” or “open fracture” because the disease status may be different and the diagnosis may be imprecise; patients with missing E/F file data (containing procedure details), height or weight data, as these variables were necessary for risk adjustment in the analysis; patients with a diagnosis of high-energy trauma (ICD-10 code: T148) to restrict the analysis to low-energy injuries; patients requiring other major trauma-related surgeries for concomitant head, chest, abdominal, spinal, or pelvic injuries on or before the day of femoral shaft fixation, to exclude severe cases related to high-energy trauma; patients admitted to the ICU or an emergency ward within the day after admission because they were considered to represent severe cases; patients who underwent surgery on or after the 8th day of admission, as prior research indicates that such extreme delays often reflect atypical clinical situations, to ensure cohort homogeneity and reduce their potential impact on the results [19]; patients who died within the first two days of admission, as this time point was set as the landmark for the analysis to mitigate immortal time bias. Additionally, patients with missing information on some hospitalization costs were excluded from the analysis only when calculating hospitalization costs.

### Exposure

The timing of surgery for femoral shaft fractures was the exposure variable in this study. Early surgery was defined as surgery performed on the day of admission or the day after admission. This definition was based on the Japanese reimbursement policy, which provides financial incentives for hip fracture surgeries performed within 48 h after injury. In cases where surgery for femoral shaft fracture was performed more than once during the hospital stay, only the first procedure was considered. Patients who underwent early surgery were categorized into the Early Surgery group, while those who underwent surgery later were classified into the Delayed Surgery group.

### Outcome

The primary outcome variable was in-hospital mortality. Secondary outcomes included in-hospital complications, length of postoperative hospital stay, and hospitalization costs. Total length of hospital stay was assessed as a supplemental outcome given the structural bias related to time to surgery. Complications were defined as diseases developed after admission and were identified using the following ICD-10 codes: deep vein thrombosis (I80.2), pulmonary embolism (I26.9), delirium (F05.0, F05.1, F05.9), pneumonia (J69.0, J85.1, J95.8, J12–J18), urinary tract infection (N10, N30.0, N30.8, N30.9, N34.1), peroneal nerve palsy (G57.3), and decubitus ulcer (L89.9). Hospitalization costs were calculated as the total of all costs related to healthcare services during hospitalization. The total cost included charges for basic inpatient care, specific inpatient care, initial consultation, laboratory tests, diagnostic imaging, medications, injections, therapeutic procedures, surgery, and medical guidance. All costs were calculated in Japanese yen (JPY).

### Statistical analysis

Baseline characteristics and outcomes are summarized for the overall cohort, as well as for the Early Surgery and Delayed Surgery groups. Continuous variables are expressed as means with standard deviations (SD) or medians with the interquartile range (IQR), and categorical variables are presented as counts and percentages. To evaluate the effect of time to surgery on clinical outcomes, we performed inverse probability of treatment weighting (IPTW) using propensity scores to reduce the influence of confounding factors [20, 21]. Propensity scores were calculated using a multivariate logistic regression model, and weighting was based on the propensity score for each patient to adjust for balance between the Early Surgery and Delayed Surgery groups.

The IPTW model included the following covariates: age, sex, body mass index, Barthel Index (BI) at admission, pre-admission living status (home or facility), holiday admission, pre-holiday admission, heart failure, cardiovascular disease, chronic obstructive pulmonary disease, diabetes mellitus, dementia, chronic renal failure, rheumatic disease, cerebrovascular disease, non-metastatic solid tumor, metastatic solid tumor, pneumonia at admission, urinary tract infection at admission, decubitus ulcer at admission, traumatic brain injury, chest injury, abdominal injury, pelvic injury, spinal injury, open fractures of other sites, other fractures, preoperative skeletal traction, preoperative skin traction, general anesthesia, blood transfusion on or before the day of surgery, preoperative antithrombotic drug use, and oral steroids use. These variables were selected based on their clinical relevance and previous reports of confounders [22]. To clearly distinguish baseline confounders from postoperative outcomes, all comorbidities used as covariates were defined as diseases recorded as “comorbidities at admission”. Given that BI at admission had missing data (14.6%), we performed multiple imputations by chained equations to impute these values, assuming that the data were missing at random [23]. We generated m = 20 complete datasets. For each of the 20 datasets, propensity scores were first estimated to create IPTW weights. The covariate balance was assessed by averaging the weighted standardized mean differences (SMD) across all 20 datasets. An SMD of 0 indicates perfect balance and a value less than 0.1 is generally considered acceptable [20, 24, 25]. Outcome analyses were then conducted on each dataset using weighted regression models that accounted for institutional clustering by hospital code. For in-hospital mortality and complications, odds ratios (ORs) and 95% confidence intervals (CIs) were estimated using logistic regression. For postoperative and total length of hospital stay and hospitalization costs, mean differences and 95% CIs were estimated using linear regression. All final estimates were derived by pooling these 20 sets of results using Rubin’s Rules [26].

### Sensitivity analysis

To reduce the effect of cases with extreme propensity scores, we trimmed the propensity scores from the entire cohort based on the 2.5th percentile of the propensity score in the Early Surgery group and the 97.5th percentile of the propensity score in the Delayed Surgery group. After recalculating the propensity scores in the trimmed cohort, we performed IPTW analysis accordingly [27, 28]. We also changed the definition of early surgery to include cases in which surgery was performed within three days of admission. The same analytical procedure was applied for this definition. Furthermore, to confirm the robustness of the results across different statistical approaches, we performed a sensitivity analysis using a multivariate regression model. This model adjusted for all covariates included in the IPTW model, utilized multiple imputation datasets, and accounted for facility clustering.

All statistical analyses were performed using R Version 4.4.2. Multiple imputation was performed using the mice package, and weighting and outcome analyses were performed using the WeightIt and Survey packages. A p-value of < 0.05 was considered statistically significant.

## Results

**Fig. 1:**
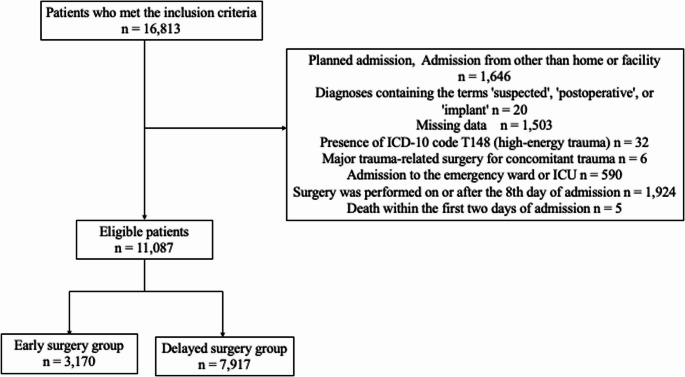
Study cohort inclusion process. Abbreviations: ICU, Intensive care unit

A total of 16,813 patients aged 65 years and over who underwent surgery for femoral shaft fractures between April 2014 and March 2023 were identified in the DPC database. Of these, 1,646 patients were excluded as a result of being transferred from another hospital, a scheduled admission or an admission route other than home or facility, 20 patients were excluded because their diagnosis included the terms suspected, postoperative, or implant, 1,503 patients had missing data, 32 patients had a diagnosis of high-energy trauma, 6 patients who underwent other major trauma-related surgeries, 590 patients were admitted to the ICU or an emergency ward within the day after admission, 1,924 patients underwent surgery on or after the 8th day of admission, and 5 patients died within the first two days of admission. After these exclusions, 11,087 patients were included in the analysis. Among them, 3,170 patients (28.6%) were assigned to the Early Surgery group, and the remaining 7,917 patients were classified into the Delayed Surgery group (Fig. 1). The hospitalization costs analysis included 10,987 patients, as 100 patients were excluded because of missing cost data. These patients were excluded only from the cost analysis, as other clinical outcomes were available.

### Baseline characteristics and covariate balance

Table 1 shows the characteristics of the overall cohort and the Early Surgery and Delayed Surgery groups before and after IPTW adjustment. The mean age of the overall cohort was 82.1 years, and 87.8% of the patients were female. The mean time to surgery in the overall cohort was 3.75 days. Compared to the Early Surgery group, the Delayed Surgery group had higher proportions of holiday admissions, pre-holiday admissions, use of skeletal traction, general anesthesia, and preoperative blood transfusions.

**Fig. 2:**
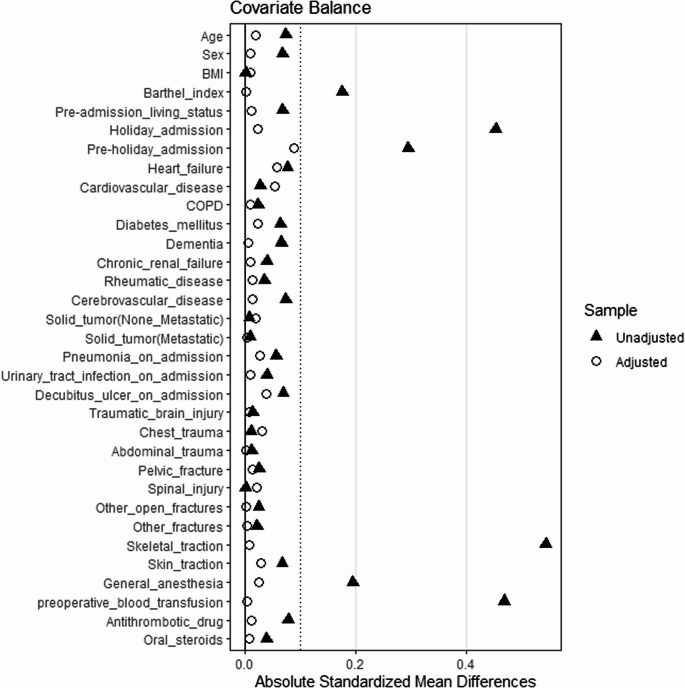
Covariate balance before and after IPTW adjustment in the overall cohort Abbreviations: IPTW, Inverse probability of treatment weighting; BMI, body mass index; COPD, chronic obstructive pulmonary disease

After IPTW adjustment, SMDs for all covariates were less than 0.1, indicating an acceptable balance between the two groups (Fig. 2). In the hospitalization costs analysis, an acceptable balance between the two groups was also observed after IPTW adjustment (Supplemental Fig. 1, Supplemental Table 1 in the Online resource). The clinical outcomes of the overall cohort and each group are shown in Table 2. The overall in-hospital mortality rate was 2.1%, and the most frequent complication was deep vein thrombosis, occurring in 5.2% of patients. Regarding discharge destinations, the overall home discharge rate was 33.9%, with 32.8% in the early surgery group and 34.4% in the delayed surgery group. The overall transfer rate to other hospitals was 48.8%, with 51.8% in the early surgery group and 47.5% in the delayed surgery group. The median length of postoperative hospital stay was 27 days in the Early Surgery group and 31 days in the Delayed Surgery group. The median hospitalization costs were 1,540,860 JPY in the Early Surgery group and 1,729,180 JPY in the Delayed Surgery group.

**Table 1:** Baseline characteristics of the overall cohort and the early surgery and delayed surgery groups, before and after IPTW adjustment

	Overall	Before IPTW			After IPTW		
		Early surgery group	Delayed surgery group	SMD	Early surgery group	Delayed surgery group	SMD
n (%)	11,087	3,170 (28.6)	7,917 (71.4)	-	10,922.6	11,105.1	-
Hospital Admission to Initial Operation (Days), mean (SD)	3.75 (1.72)	1.74 (0.44)	4.55 (1.34)	-	-	-	-
Age, years, mean (SD)	82.12 (8.09)	81.69 (8.16)	82.29 (8.06)	0.073	82.29 (8.24)	82.15 (8.09)	0.018
Female, *n* (%)	9,736 (87.8)	2,733 (86.2)	7,003 (88.5)	0.067	9,550.5 (87.4)	9,740.6 (87.7)	0.008
BMI, kg/m^2^, mean (SD)	22.26 (3.98)	22.26 (3.84)	22.26 (4.03)	< 0.001	22.24 (3.82)	22.27 (4.03)	0.008
Barthel Index (BI) at admission, mean (SD)	16.07 (27.7)	17.95 (29.8)	15.32 (26.8)	0.093	16.1 (27.8)	16.1 (27.5)	< 0.001
Pre-admission living status: Facility, *n *(%)	1,708 (15.4)	434 (13.7)	1,274 (16.1)	0.067	1,644.5 (15.1)	1,714.8 (15.4)	0.011
Holiday admission, *n* (%)	3,422 (30.9)	535 (16.9)	2,887 (36.5)	0.454	3,470.1 (31.8)	3,421.8 (30.8)	0.022
Pre-holiday admission, *n* (%)	1,870 (16.9)	301 (9.5)	1,569 (19.8)	0.295	1,486.6 (13.6)	1,851.58 (16.7)	0.088
Heart failure, *n* (%)	757 (6.8)	174 (5.5)	583 (7.4)	0.077	909.2 (8.3)	766.7 (6.9)	0.058
Cardiovascular disease, *n* (%)	825 (7.4)	220 (6.9)	605 (7.6)	0.027	974.5 (8.9)	837.0 (7.5)	0.053
COPD, *n* (%)	97 (0.9)	23 (0.7)	74 (0.9)	0.023	83.7 (0.8)	94.2 (0.8)	0.009
Diabetes mellitus, *n* (%)	1,944 (17.5)	502 (15.8)	1,442 (18.2)	0.063	2,028.4 (18.6)	1,970.4 (17.7)	0.022
Dementia, *n* (%)	1,444 (13.0)	364 (11.5)	1,080 (13.6)	0.065	1,440.7 (13.2)	1,448.9 (13.0)	0.004
Chronic renal failure, *n* (%)	51 (0.5)	9 (0.3)	42 (0.5)	0.039	57.4 (0.5)	52.2 (0.5)	0.009
Rheumatic disease, *n* (%)	426 (3.8)	107 (3.4)	319 (4.0)	0.035	447.8 (4.1)	429.8 (3.9)	0.012
Cerebrovascular disease, *n* (%)	854 (7.7)	201 (6.3)	653 (8.2)	0.073	804.6 (7.4)	856.3 (7.7)	0.013
Non-metastatic solid tumor, *n* (%)	600 (5.4)	168 (5.3)	432 (5.5)	0.007	636.5 (5.8)	601.1 (5.4)	0.018
Metastatic solid tumor, *n* (%)	194 (1.7)	58 (1.8)	136 (1.7)	0.008	184.6 (1.7)	192.3 (1.7)	0.003
Pneumonia at admission, *n* (%)	52 (0.5)	7 (0.2)	45 (0.6)	0.055	32.4 (0.3)	51.2 (0.5)	0.026
Urinary tract infection at admission, *n* (%)	60 (0.5)	11 (0.3)	49 (0.6)	0.039	51.9 (0.5)	60.1 (0.5)	0.010
Decubitus ulcer at admission, *n* (%)	76 (0.7)	10 (0.3)	66 (0.8)	0.069	43.4 (0.4)	75.3 (0.7)	0.037
Traumatic brain injury, *n* (%)	23 (0.2)	8 (0.3)	15 (0.2)	0.013	20.1(0.2)	24.1(0.2)	0.007
Chest injury, *n* (%)	57 (0.5)	18 (0.6)	39 (0.5)	0.010	81.4 (0.7)	59.0 (0.5)	0.030
Abdominal injury, *n* (%)	5 (0.0)	2 (0.1)	3 (0.0)	0.011	5.7 (0.1)	5.9 (0.1)	< 0.001
Pelvic injury, *n* (%)	37 (0.3)	14 (0.4)	23 (0.3)	0.025	29.4 (0.3)	38.2 (0.3)	0.012
Spinal injury, *n* (%)	77 (0.7)	22 (0.7)	55 (0.7)	< 0.001	96.4 (0.9)	78.6 (0.7)	0.021
Other open fractures, *n* (%)	11 (0.1)	5 (0.2)	6 (0.1)	0.024	8.39 (0.1)	8.65 (0.1)	< 0.001
Other fractures, *n* (%)	651 (5.9)	175 (5.5)	476 (6.0)	0.021	665.2 (6.1)	652.3 (5.9)	0.003
Skeletal traction, *n* (%)	5,013 (45.2)	851 (26.8)	4,162 (52.6)	0.545	4,892.5 (44.8)	5,006.1 (45.1)	0.006
Skin traction, *n* (%)	1,211 (10.9)	300 (9.5)	911 (11.5)	0.067	1,088.0 (10.0)	1,203.8 (10.8)	0.029
General anesthesia, *n* (%)	7,656 (69.1)	1,983 (62.6)	5,673 (71.7)	0.195	7,691.1 (70.4)	7,697.8 (69.3)	0.023
Preoperative blood transfusion, *n* (%)	1,997 (18.0)	206 (6.5)	1,791 (22.6)	0.470	1,952.2 (17.9)	1,997.0 (18.0)	0.003
Antithrombotic drug, *n* (%)	4,552 (41.1)	1,215 (38.3)	3,337 (42.1)	0.078	4,545.8 (41.6)	4,565.7 (41.1)	0.010
Oral steroids, *n* (%)	654 (5.9)	167 (5.3)	487 (6.2)	0.038	665.2 (6.1)	658.4 (5.9)	0.007

*IPTW *Inverse probability of treatment weighting, *SMD *Standard mean difference, *SD * Standard deviation, *BMI *body mass index, *COPD* chronic obstructive pulmonary disease

Barthel Index in “Before IPTW” is based on non-missing cases (*n* = 9,472, 85.4%). The “After IPTW” value for this variable is the pooled estimate derived from multiple imputation

n in “After IPTW” columns represents the effective sample size after weighting

**Table 2:** Outcomes of the overall cohort and early surgery and delayed surgery groups

	Overall	Early surgery group	Delayed surgery group
In-hospital mortality, *n* (%)	238 (2.1)	64 (2.0)	174 (2.2)
Deep vein thrombosis, *n* (%)	580 (5.2)	152 (4.8)	428 (5.4)
Pulmonary embolism, *n* (%)	53 (0.5)	14 (0.4)	39 (0.5)
Pneumonia, *n* (%)	230 (2.1)	53 (1.7)	177 (2.2)
Urinary tract infection, *n* (%)	361 (3.3)	73 (2.3)	288 (3.6)
Delirium, *n* (%)	174 (1.6)	47 (1.5)	127 (1.6)
Decubitus ulcer, *n* (%)	166 (1.5)	29 (0.9)	137 (1.7)
Peroneal nerve palsy, *n* (%)	14 (0.1)	3 (0.1)	11 (0.1)
Home discharge	3,761 (33.9)	1,040 (32.8)	2,721 (34.4)
Transfer to other hospitals	5,405 (48.8)	1,641 (51.8)	3,764 (47.5)
Length of postoperative hospital stay, median [IQR]	29 [18–54]	27 [17–47]	31 [19–57]
Hospitalization costs (1000 JPY), median [IQR]	1,670.63 [1,342.00–2,356.73]	1,540.86 [1,265.68-2,167.88]	1,729.18 [1,373.46-2,427.41]

*IQR *Interquartile range, *JPY* Japanese yen

### Outcome analysis adjusted by IPTW

Table 3 shows the IPTW-adjusted estimates for the clinical outcomes. The OR for in-hospital mortality in the Early Surgery group compared with the Delayed Surgery group was 0.96 (95% CI, 0.66–1.41), which was not statistically significant. Among in-hospital complications, pneumonia (OR, 0.64; 95% CI, 0.44–0.94) and urinary tract infection (OR, 0.66; 95% CI, 0.48–0.90) were significantly less frequent in the Early Surgery group, but the incidence of other complications did not significantly differ between the groups. The length of postoperative hospital stay was significantly shorter in the Early Surgery group compared with the Delayed Surgery group, with a mean difference of 5.37 days (95% CI, 3.56–7.19). Hospitalization costs were significantly lower in the Early Surgery group, with a mean difference of 150,380 JPY (95% CI, 93,270–207,490) compared with the Delayed Surgery group. Analysis of total length of hospital stay, treated as a supplemental outcome, is presented in the Supplementary Materials (Supplementary Table 2).

**Table 3:** IPTW-adjusted odds ratios and mean differences for the clinical outcomes

Outcome	IPTW analysis	
	Estimate [95% CI]	*P*-value
Categorical variables	Odds ratio	
In-hospital mortality	0.96 [0.66–1.41]	0.835
Deep vein thrombosis	0.98 [0.73–1.32]	0.891
Pulmonary embolism	0.60 [0.32–1.14]	0.117
Pneumonia	0.64 [0.44–0.94]	0.021
Urinary tract infection	0.66 [0.48–0.90]	0.009
Delirium	1.11 [0.73–1.65]	0.653
Decubitus ulcer	0.63 [0.34–1.18]	0.149
Peroneal nerve palsy	0.40 [0.10–1.61]	0.197
Continuous variables	Mean differences	
Length of postoperative hospital stay	5.37 [3.56–7.19]	< 0.001
Hospitalization costs (1000 JPY)	150.38 [93.27-207.49]	< 0.001

*IPTW *Inverse probability of treatment weighting, *CI *confidence interval, *JPY *Japanese yen

### Sensitivity analysis

In the sensitivity analysis with trimming by propensity scores, the covariate balance between the Early Surgery and Delayed Surgery groups after IPTW adjustment was acceptable (Supplemental Fig. 2, Supplemental Table 3). In this cohort, there was no statistically significant difference in in-hospital mortality, and shorter postoperative hospital stays and lower hospitalization costs remained statistically significant. Regarding complications, the findings differed from the main analysis: the Early Surgery group showed a significantly lower ORs of urinary tract infection and decubitus ulcers, while the association with pneumonia was not statistically significant (Supplemental Table 4).

When early surgery was redefined as surgery performed within three days of admission, 5,423 patients (48.9% of the total cohort) were categorized into the Early Surgery group. The covariate balance between the two groups after IPTW adjustment was acceptable (Supplemental Fig. 3, Supplemental Table 5). In this cohort, there was no statistically significant difference in in-hospital mortality, and shorter postoperative hospital stays and lower hospitalization costs remained statistically significant. Regarding complications, a significantly lower OR was observed for urinary tract infection (Supplemental Table 6).

Furthermore, the sensitivity analysis using a multivariate regression model also showed no statistically significant difference in in-hospital mortality. In this cohort, the Early Surgery group had a significantly lower ORs of urinary tract infection and decubitus ulcers. Additionally, shorter postoperative hospital stays and lower hospitalization costs were observed in the Early Surgery group (Supplemental Table 7).

## Discussion

We used a nationwide administrative database in Japan to evaluate the effect of time to surgery on clinical outcomes in older adults with femoral shaft fractures. Early surgery, defined as surgery performed on the day of admission or the day after admission, was not significantly associated with in-hospital mortality but was significantly associated with a lower incidence of pneumonia and urinary tract infections, as well as shorter postoperative hospital stays and lower hospitalization costs.

Several studies have reported mortality and complication rates in patients with femoral shaft fractures, though their findings varied. One study reported a 30-day in-hospital mortality rate of 13.2% in older adults with femoral shaft fractures, almost double that of hip fractures [29]. Femoral shaft fractures in older adults have a one-year mortality rate of 25% and an overall in-hospital complication rate of 41.5% [2]. In contrast, patients with isolated femoral shaft fractures have a 30-day mortality rate of 2.7% and prevalences of deep vein thrombosis and pulmonary embolism of 1.3% and 1.1%, respectively [30]. Another study using a nationwide database in Japan reported an in-hospital mortality rate of 2% for hip fractures [31]. In this study, the overall in-hospital mortality rate was 2.2%, which is lower than previously reported rates for femoral shaft fractures and comparable to those for hip fractures in Japan. One possible explanation for the relatively low rates of mortality and complications observed in this study is the prolonged median length of postoperative hospital stay of 29 days, which may have allowed sufficient time for comprehensive care and recovery. Additionally, our exclusion of critically ill patients requiring ICU or emergency ward admission, as well as those undergoing surgery more than 8 days after the time of admission, may have contributed to these results.

In this study, the mean time from admission to surgery was 3.75 days, and 28.6% of patients underwent surgery on the day of admission or the day after. This proportion is lower than that reported from other countries. A review of patients with multiple injuries found that 70.4% underwent surgery within 24 h. In a study of isolated femoral shaft fractures, 92.7% of patients underwent surgery within three days of admission [10, 30]. In Japan, the proportion of patients undergoing early surgery for hip fracture is low, with 6.8% of patients undergoing surgery on the day of admission, 25.9% by the next day, and 39.4% within three days, consistent with the trend observed in our study [31]. These results may indicate limitations in the healthcare delivery system for providing early surgery for fractures in Japan, as well as limited awareness among healthcare providers regarding the importance of early surgical intervention.

Regarding the relationship between time to surgery and mortality, previous studies (including severe injury and high-energy trauma) report that delayed surgery for femoral shaft fractures is associated with increased postoperative mortality [6–8]. In contrast, a study focusing on isolated femoral shaft fractures found no association between delayed surgery and postoperative mortality, which aligns with our findings as we excluded severe and high-energy trauma cases [30]. One possible explanation for the lack of association between time to surgery and in-hospital mortality in this study is that delays were partly the result of the need for preoperative management of comorbidities and the overall patient condition, which may have contributed to reducing in-hospital mortality. In addition, perioperative and postoperative care, critical determinants of mortality, were likely adequately provided within the Japanese healthcare system, which may have diminished the relative impact of surgical timing on in-hospital mortality.

Many studies report that early surgery is associated with a lower incidence of complications. Delayed surgery for patients with severe injuries or high-energy trauma is associated with increased rates of acute respiratory distress syndrome, pulmonary complications, decubitus ulcers, and deep vein thrombosis, whereas early surgery is associated with fewer infections and thromboembolic events [8–10]. Although few studies have specifically examined complications following early surgery for femoral shaft fractures in older adults, studies of hip fracture show that surgery performed more than 48 h after injury or admission is associated with a higher risk of urinary tract infection and aspiration pneumonia, which is consistent with our findings [32–34]. One possible explanation for the reduced incidence of pneumonia and urinary tract infections in the Early Surgery group may be that earlier surgical intervention may have facilitated early mobilization, thereby decreasing the risk of these complications.

Early surgery is often associated with a shorter length of hospital stay, and our results are consistent with previous research [35–37]. This may be attributed to the fact that early surgery allowed for a more rapid recovery and an earlier discharge. Additionally, patients in the Early Surgery group may have had more stable baseline conditions, which facilitated smoother transitions out of the acute care setting.

Regarding hospitalization costs, studies on hip fractures and femoral fractures have reported that early surgery is associated with reduced treatment costs [35, 38–41]. Our findings are consistent with these prior reports. In Japan’s healthcare payment system (DPC/PDPS), longer hospital stays and higher complication rates result in increased total hospitalization costs. Therefore, a reduction in complications and shorter hospital stays may have directly affected cost savings.

The British Orthopaedic Association guidelines recommend that all surgeries for older and frail patients with fractures be performed within 36 h of admission, following the same principles as for treating hip fractures [42]. Previous studies also suggest that older adults with femoral shaft fractures should be managed similarly to those with hip fractures [29, 30]. In line with these recommendations, our findings show that early surgery is associated with fewer complications and shorter postoperative hospital stays, and reduced hospitalization costs, demonstrating the benefits of early surgery for improving patient outcomes and promoting the efficient use of medical resources.

Our sensitivity analyses reinforced the outcomes of the primary analysis. Across all sensitivity analyses, no association was found between early surgery and in-hospital mortality. Furthermore, shorter postoperative hospital stays and lower hospitalization costs remained consistently significant in these models. The findings for postoperative complications also supported the main analysis, although the specific significant complications varied slightly across models. In the sensitivity analysis with trimming by propensity scores, early surgery was associated with significantly lower odds of urinary tract infection and decubitus ulcers. In the analysis redefining early surgery as surgery performed within 3 days of admission, the odds of urinary tract infection were also significantly lower in the Early Surgery group. Multivariate regression analysis showed lower odds of urinary tract infection and decubitus ulcers. Collectively, these findings support the robustness of our primary results and suggest that early surgical intervention may reduce postoperative complications, thereby contributing to more efficient utilization of healthcare resources. Importantly, no adverse outcomes were associated with early intervention. Given that time to surgery is a modifiable factor, early surgical intervention should be recommended for older adults with femoral shaft fractures, in accordance with the approach for hip fractures.

This study had several limitations. The most important limitation is the potential influence of unmeasured confounding factors that are not captured in the database. For instance, variables such as the method of internal fixation, laboratory data, patients’ social background, ASA Physical Status, implementation of interdisciplinary care, and hospital-specific healthcare systems could not be accounted for in our analyses. Although IPTW was applied to adjust for major confounding factors and improve the balance between the groups, the presence of residual confounding factors cannot be fully ruled out, and the results should be interpreted with caution. Nevertheless, it is unlikely that unmeasured confounding factors would be strong enough to account for the observed associations between surgical timing and clinical outcomes. Secondly, the database used in this study only recorded outcomes up to hospital discharge, making it impossible to evaluate long-term clinical or functional outcomes. Further research is needed to evaluate the long-term effects of early surgery. Thirdly, time to surgery was defined as the interval from hospital admission to surgery, which may not accurately reflect the actual time from injury to surgery. However, as most patients with femoral shaft fractures are thought to go to the hospital soon after injury, we consider that the impact of this discrepancy is likely to be limited. Fourth, we could not completely exclude patients with high-energy trauma and severe injuries, and the Injury Severity Score (ISS)—a widely used indicator for quantifying the severity of multiple trauma—could not be obtained from the database. To mitigate this limitation, we excluded patients with diagnoses of high-energy trauma, those admitted to the ICU or emergency ward immediately after admission, and those who required major trauma-related surgery for concomitant injuries on or before the day of femoral shaft fixation. Furthermore, we adjusted for concomitant injuries to the head, chest, abdomen, spine, and pelvis, open fractures of other sites, and other fractures as covariates in the IPTW model. However, we were unable to exclude patients based on procedures potentially indicating severe injury—such as central venous catheter insertion, tracheal intubation, mechanical ventilation, and early vasopressor use—because the database lacks precise time-stamps to differentiate between initial resuscitation and perioperative management.　Consequently, despite efforts to minimize bias, it is possible that not all severe polytrauma cases were completely captured. Finally, the interpretation of our outcomes, particularly length of postoperative hospital stay and hospitalization costs, requires caution. In the Japanese healthcare system, subacute rehabilitation following acute treatment and discharge or transfer coordination to rehabilitation hospitals are typically included within the same hospitalization period. Therefore, the observed length of stay and costs in this study strongly reflect not only the pure clinical effects of timing surgery, but also the impact of these non-clinical and social delays related to discharge destinations. Because of this specific background of the Japanese healthcare system, it may be difficult to generalize the results, particularly regarding length of stay and costs, to healthcare systems in other countries. Despite these limitations, this is the first study to evaluate the impact of early surgery for femoral shaft fractures in older adults, excluding severe or high-energy trauma cases, using a nationwide multicenter database. The use of IPTW enhances the internal validity of the findings, and the use of a large-scale national dataset supports their robustness and reliability.

## Conclusion

This study evaluated the effect of early surgery on clinical outcomes in older adults with femoral shaft fractures. Early surgery, defined as surgery performed on the day of admission or the day after admission, was not associated with a significant reduction in in-hospital mortality. However, it was associated with reduced risks of certain complications, as well as shorter postoperative hospital stays and lower hospitalization costs. These findings suggest that early surgical intervention for older adults with femoral shaft fractures may be beneficial in reducing postoperative complications, length of stay, and hospitalization costs, thereby contributing to more efficient utilization of healthcare resources. Further research is needed to confirm these findings and to assess the long-term outcomes of early surgery, including mortality rates and functional recovery.

## Supplementary Information

Below is the link to the electronic supplementary material.

Supplementary Material 1 (PDF 324 KB)

## Data Availability

The Japanese government has made a law “Act on the Protection of Personal Information”, which restricts the use of personal information. Our dataset includes potentially sensitive information, such as diagnosis and medical histories regarded as “Special care-required personal information” specified by the above law. And if our dataset would be combined with other datasets, it can be linked to personal identification. Moreover, our datasets were collected from each hospital within the bounds of the ethical procedures of academic research and the data use were limited to the researchers listed on the research plan submitted in advance to the Ethics Committee. Therefore, our data are not permitted to release publicly. The Japanese ethical guideline “Ethical Guidelines for Medical and Health Research Involving Human Subjects” has imposed these restrictions in consistency with the law “Act on the Protection of Personal Information”. Requests to access the data should be submitted to Kyoto University Graduate School of Medicine/Faculty General Affairs Division, Research Promotion Section.
