# Supplementary material for: The effect of time to surgery on clinical outcomes and hospitalization costs in older adults with femoral shaft fractures: A nationwide retrospective cohort study in Japan
Source: Eur J Trauma Emerg Surg. 2026 Feb 23;52(1):53. doi: 10.1007/s00068-026-03106-7 (PMC12929321; doi:10.1007/s00068-026-03106-7)
Supplement: Supplementary file 1 — Supplementary Material 1 (PDF 324 KB) [file 68_2026_3106_MOESM1_ESM.pdf]

The Effect of Time to Surgery on Clinical Outcomes and Hospitalization Costs in Older Adults with Femoral Shaft Fractures: A Nationwide Retrospective Cohort Study in Japan

Authors

Gaku Gondo[1], Daisuke Takada[1,2], Susumu Kunisawa[1], Kiyohide Fushimi[3], Yuichi Imanaka[4,1]

Affiliations

1. Department of Healthcare Economics and Quality Management, School of Public Health, Graduate School of Medicine, Kyoto University, Kyoto, Japan.
2. Department of Food Science and Nutrition, Faculty of Human Life and Science, Doshisha Women's college of Liberal Arts, Kyoto, Japan.
3. Department of Health Policy and Informatics Institute of Science Tokyo Graduate School of Medical and Dental Sciences, Tokyo, Japan.
4. Department of Health Security System, Centre for Health Security Graduate School of Medicine, Kyoto University, Kyoto, Japan.

Corresponding author

Yuichi Imanaka

Email: imanaka-y@umin.net.

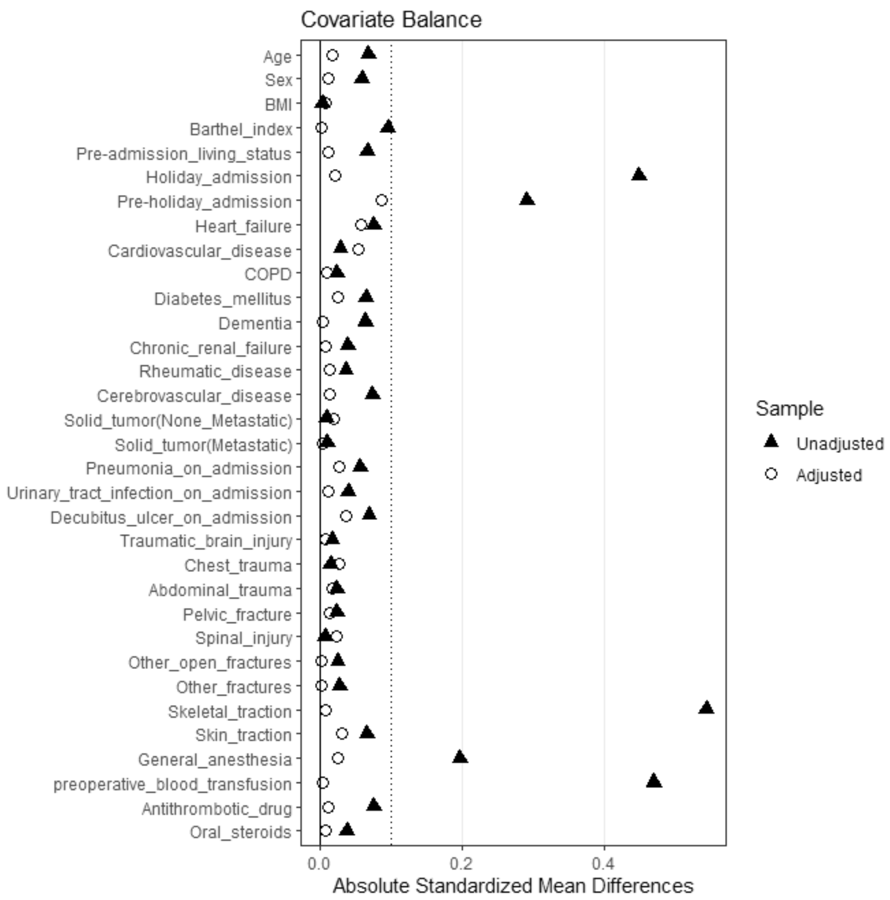

Supplemental Fig. 1 Covariate balance before and after IPTW adjustment in the hospitalization cost analysis cohort. Abbreviations: IPTW, Inverse probability of treatment weighting; BMI, body mass index; COPD, chronic obstructive pulmonary disease

Supplemental Table 1 Baseline characteristics of the overall cohort and the Early Surgery and Delayed Surgery groups, before and after IPTW adjustment in the hospitalization cost analysis Cohort

	Before IPTW				After IPTW		
	Overall	Early surgery group	Delayed surgery group	SMD	Early surgery group	Delayed surgery group	SMD
n	10,987	3,130	7,857	-	10,822.6	11,004.2	-
Age, years, mean (SD)	82.21 (8.05)	81.82 (8.11)	82.36 (8.03)	0.067	82.37 (8.20)	82.2 (8.05)	0.017
Female, n (%)	9,691 (88.2)	2,718 (86.8)	6,973 (88.7)	0.058	9,501.8 (87.8)	9,697.1 (88.1)	0.010
BMI, kg/m^2, mean (SD)	22.25 (3.98)	22.24 (3.84)	22.25 (4.04)	0.003	22.2 (3.82)	22.3 (4.04)	0.007

Barthel Index (BI) at admission, mean (SD)	16.00 (27.62)	17.93 (29.76)	15.24 (26.69)	0.095	16.03 (27.69)	16.03 (27.39)	<0.001
Pre-admission living status: Facility, n (%)	1,708 (15.5)	434 (13.9)	1,274 (16.2)	0.066	1,641.4 (15.2)	1,714.7 (15.6)	0.012
Holiday admission, n (%)	3,398 (30.9)	533 (17.0)	2,865 (36.5)	0.450	3,437.3 (31.8)	3,397.3 (30.9)	0.021
Pre-holiday admission, n (%)	1,856 (16.9)	300 (9.6)	1,556 (19.8)	0.292	1,479.0 (13.7)	1,837.6 (16.7)	0.087
Heart failure, n (%)	755 (6.9)	174 (5.6)	581 (7.4)	0.075	903.4 (8.3)	764.5 (6.9)	0.057
Cardiovascular disease, n (%)	823 (7.5)	218 (7.0)	605 (7.7)	0.028	971.6 (9.0)	835.1 (7.6)	0.053
COPD, n (%)	97 (0.9)	23 (0.7)	74 (0.9)	0.023	83.5 (0.8)	94.2 (0.9)	0.009
Diabetes mellitus, n (%)	1,923 (17.5)	494 (15.8)	1,429 (18.2)	0.064	2,015.2 (18.6)	1,951.0 (17.7)	0.024
Dementia, n (%)	1,443 (13.1)	364 (11.6)	1,079 (13.7)	0.063	1,436.2 (13.3)	1,447.5 (13.2)	0.003
Chronic renal failure, n (%)	51 (0.5)	1 (0.1)	42 (0.5)	0.039	56.7 (0.5)	52.2 (0.5)	0.008
Rheumatic disease, n (%)	425 (3.9)	106 (3.4)	319 (4.1)	0.036	446.6 (4.1)	428.4 (3.9)	0.012
Cerebrovascular disease, n (%)	851 (7.7)	200 (6.4)	651 (8.3)	0.073	801.9 (7.4)	853.4 (7.8)	0.013
Non-metastatic solid tumor, n (%)	597 (5.4)	166 (5.3)	431 (5.5)	0.008	633.9 (5.9)	597.9 (5.4)	0.019
Metastatic solid tumor, n (%)	194 (1.8)	58 (1.9)	136 (1.7)	0.009	185.0 (1.7)	192.2 (1.7)	0.003
Pneumonia at admission, n (%)	52 (0.5)	7 (0.2)	45 (0.6)	0.055	32.3 (0.3)	51.2 (0.5)	0.026
Urinary tract infection at admission, n (%)	60 (0.5)	11 (0.4)	49 (0.6)	0.039	51.4 (0.5)	60.1 (0.5)	0.010
Decubitus ulcer at admission, n (%)	76 (0.7)	10 (0.3)	66 (0.8)	0.069	44.4 (0.4)	75.3 (0.7)	0.036
Traumatic brain injury, n (%)	22 (0.2)	8 (0.3)	14 (0.2)	0.017	19.6 (0.2)	23.2 (0.2)	0.006
Chest injury, n (%)	52 (0.5)	17 (0.5)	35 (0.4)	0.014	72.7 (0.7)	53.5 (0.5)	0.026
Abdominal injury, n (%)	2 (0.0)	0 (0.0)	2 (0.0)	0.023	0.0 (0.0)	2.0 (0.0)	0.016
Pelvic injury, n (%)	35 (0.3)	13 (0.4)	22 (0.3)	0.023	26.5 (0.2)	34.5 (0.3)	0.012

Spinal injury, n (%)	74 (0.7)	20 (0.6)	54 (0.7)	0.006	93.0 (0.9)	75.1 (0.7)	0.022
Other open fractures, n (%)	11 (0.1)	5 (0.2)	6 (0.1)	0.024	8.3 (0.1)	8.6 (0.1)	<0.001
Other fractures, n (%)	634 (5.8)	167 (5.3)	467 (5.9)	0.026	620.6 (5.7)	635.3 (5.8)	0.002
Skeletal traction, n (%)	4,954 (45.1)	836 (26.7)	4,118 (52.4)	0.545	4,835.8 (44.7)	4,947.5 (45.0)	0.006
Skin traction, n (%)	1,211 (11.0)	300 (9.6)	911 (11.6)	0.065	1,087.0 (10.0)	1,203.5 (10.9)	0.029
General anesthesia, n (%)	7,586 (69.0)	1,955 (62.5)	5,631 (71.7)	0.197	7,616.4 (70.4)	7,624.4 (69.3)	0.023
Preoperative blood transfusion, n (%)	1,993 (18.1)	205 (6.5)	1,788 (22.8)	0.471	1,948.4 (18.0)	1,993.1 (18.1)	0.003
Antithrombotic drug, n (%)	4,532 (41.3)	1,209 (38.6)	3,323 (42.3)	0.075	4,521.3 (41.8)	4,543.3 (41.3)	0.010
Oral steroids, n (%)	650 (5.9)	166 (5.3)	484 (6.2)	0.037	661.5 (6.1)	654.2 (5.9)	0.007

Abbreviations: IPTW, Inverse probability of treatment weighting; SMD, Standard mean difference; SD, Standard deviation; BMI, body mass index; COPD, chronic obstructive pulmonary disease

Barthel Index in "After IPTW" is the pooled estimate derived from multiple imputation

n in "After IPTW" columns represents the effective sample size after weighting

Supplemental Table 2 IPTW-adjusted mean differences of total length of hospital stay using the main analysis methodology

Outcome	Estimate Mean differences [95% CI]	P-value
Total length of hospital stay	8.10 [6.28-9.92]	<0.001

Abbreviations: IPTW, Inverse probability of treatment weighting; CI, confidence interval

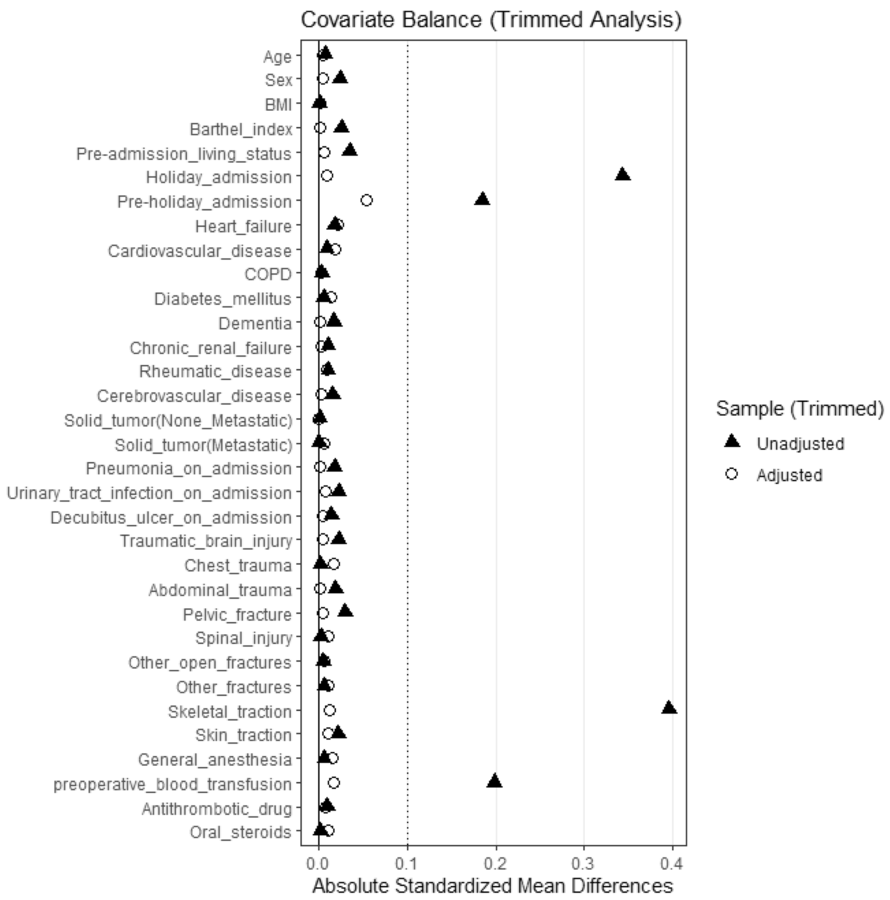

Supplemental Fig. 2 Covariate balance before and after IPTW adjustment in the trimmed cohort

Abbreviations: IPTW, Inverse probability of treatment weighting; BMI, body mass index; COPD, chronic obstructive pulmonary disease

Supplemental Table 3 Baseline characteristics of the overall cohort and the Early Surgery and Delayed Surgery groups, before and after IPTW adjustment in the trimmed cohort

	Before IPTW				After IPTW		
	Overall	Early surgery group	Delayed surgery group	SMD	Early surgery group	Delayed surgery group	SMD
n	8,763.9	2,621.2	6,142.7	-	8,683.1	8,783.7	-
Age, years, mean (SD)	81.85 (8.01)	81.91 (8.12)	81.83 (7.96)	0.010	81.89 (8.12)	81.87 (7.98)	0.002
Female, n (%)	7,719.2 (88.1)	2,296.4 (87.6)	5,422.8 (88.3)	0.021	7,627.2 (87.8)	7,733.1 (88.0)	0.006
BMI, kg/m^2, mean (SD)	22.30 (3.96)	22.30 (3.86)	22.31 (4.00)	0.002	22.32 (3.83)	22.32 (4.02)	0.001
Barthel Index (BI) at admission, mean (SD)	16.38 (27.83)	16.84 (28.50)	16.18 (27.54)	0.024	16.45 (28.12)	16.40 (27.68)	0.002

Pre-admission living status: Facility, n (%)	1,253.7 (14.3)	396.1 (15.1)	857.5 (14.0)	0.033	1,256.0 (14.5)	1,257.0 (14.3)	0.004
Holiday admission, n (%)	2,524.8 (28.8)	487.6 (18.6)	2,037.2 (33.2)	0.337	2,531.5 (29.2)	2,522.4 (28.7)	0.010
Pre-holiday admission, n (%)	1,331.3 (15.2)	283.0 (10.8)	1,048.3 (17.1)	0.182	1,139.4 (13.1)	1,315.2 (15.0)	0.054
Heart failure, n (%)	532.6 (6.1)	152.1 (5.8)	380.4 (6.2)	0.016	586.2 (6.8)	541.2 (6.2)	0.025
Cardiovascular disease, n (%)	658.5 (7.5)	193.4 (7.4)	465.1 (7.6)	0.007	702.3 (8.1)	667.7 (7.6)	0.018
COPD, n (%)	71.5 (0.8)	21.0 (0.8)	50.5 (0.8)	0.002	66.7 (0.8)	69.2 (0.8)	0.002
Diabetes mellitus, n (%)	1,464.4 (16.7)	441.6 (16.8)	1,022.9 (16.7)	0.007	1,511.4 (17.4)	1,478.1 (16.8)	0.016
Dementia, n (%)	1,033.7 (11.8)	320.1 (12.2)	713.5 (11.6)	0.016	1,018.0 (11.7)	1,034.6 (11.8)	0.002
Chronic renal failure, n (%)	30.2 (0.3)	8.0 (0.3)	22.2 (0.4)	30.2 (0.3)	31.9 (0.4)	31.1 (0.4)	0.002
Rheumatic disease, n (%)	302.2 (3.4)	94.4 (3.6)	207.8 (3.4)	0.012	316.5 (3.6)	303.0 (3.4)	0.011
Cerebrovascular disease, n (%)	596.3 (6.8)	186.3 (7.1)	410.6 (6.7)	0.018	599.5 (6.9)	600.1 (6.8)	0.003
Non-metastatic solid tumor, n (%)	474.0 (5.4)	140.3 (5.4)	333.7 (5.4)	0.003	469.6 (5.4)	473.2 (5.4)	0.001
Metastatic solid tumor, n (%)	157.1 (1.8)	47.0 (1.8)	110.1 (1.8)	<0.001	146.6 (1.7)	154.5 (1.8)	0.005
Pneumonia at admission, n (%)	29.8 (0.3)	7.0 (0.3)	22.8 (0.4)	0.018	27.7 (0.3)	29.2 (0.3)	0.002
Urinary tract infection at admission, n (%)	43.3 (0.5)	10.0 (0.4)	33.3 (0.5)	0.024	37.7 (0.4)	43.1 (0.5)	0.008
Decubitus ulcer at admission, n (%)	39.0 (0.4)	10.0 (0.4)	29.0 (0.5)	0.014	37.1 (0.4)	38.7 (0.4)	0.002
Traumatic brain injury, n (%)	18.9 (0.2)	8.0 (0.3)	10.9 (0.2)	0.026	17.3 (0.2)	19.0 (0.2)	0.003
Chest injury, n (%)	40.0 (0.5)	12.0 (0.5)	28.0 (0.5)	<0.001	48.8 (0.6)	40.7 (0.5)	0.015
Abdominal injury, n (%)	0.0 (0.0)	0.0 (0.0)	0.0 (0.0)	0.000	0.00 (0.0)	0.00 (0.0)	0.000
Pelvic injury, n (%)	26.9 (0.3)	10.9 (0.4)	16.0 (0.3)	0.026	21.5 (0.2)	24.5 (0.3)	0.005
Spinal injury, n (%)	54.1 (0.6)	15.1 (0.6)	39.0 (0.6)	0.008	66.1 (0.8)	55.2 (0.6)	0.017

Other open fractures, n (%)	9.0 (0.1)	3.0 (0.1)	6.0 (0.1)	0.005	6.3 (0.1)	8.1 (0.1)	0.006
Other fractures, n (%)	495.7 (5.7)	145.6 (5.6)	350.1 (5.7)	0.006	513.4 (5.9)	498.2 (5.7)	0.010
Skeletal traction, n (%)	3,744.2 (42.7)	776.1 (29.6)	2,968.1 (48.3)	0.391	3,647.6 (42.0)	3,736.2 (42.5)	0.011
Skin traction, n (%)	1,023.6 (11.7)	293.9 (11.2)	729.8 (11.9)	0.021	970.1 (11.2)	1,015.9 (11.6)	0.012
General anesthesia, n (%)	6,184.3 (70.6)	1,845.3 (70.4)	4,339.0 (70.6)	0.005	6,196.8 (71.4)	6,206.9 (70.7)	0.015
Preoperative blood transfusion, n (%)	856.0 (9.8)	155.4 (5.9)	700.6 (11.4)	0.196	890.7 (10.3)	857.0 (9.8)	0.015
Antithrombotic drug, n (%)	3,550.7 (40.5)	1,054.2 (40.2)	2,496.5 (40.6)	0.009	3,488.2 (40.2)	3,559.4 (40.5)	0.007
Oral steroids, n (%)	492.2 (5.6)	148.8 (5.7)	343.5 (5.6)	0.004	512.0 (5.9)	495.4 (5.6)	0.011

Abbreviations: IPTW, Inverse probability of treatment weighting; SMD, Standard mean difference; SD, Standard deviation; BMI, body mass index; COPD, chronic obstructive pulmonary disease

n in "After IPTW" columns represents the effective sample size after weighting

Supplemental Table 4 IPTW-adjusted odds ratios and mean differences of the clinical outcomes in the trimmed cohort

Outcome	IPTW analysis	
	Estimate [95% CI]	P-value
Categorical variables	Odds ratio	
In-hospital mortality	0.91 [0.62-1.33]	0.625
Deep vein thrombosis	0.91 [0.70-1.18]	0.468
Pulmonary embolism	0.62 [0.38-1.28]	0.193
Pneumonia	0.63 [0.44-0.91]	0.014
Urinary tract infection	0.73 [0.52-1.02]	0.069
Delirium	1.29 [0.85-1.97]	0.233
Decubitus ulcer	0.57 [0.34-0.98]	0.042
Peroneal nerve palsy	0.89 [0.22-3.55]	0.868
Continuous variables	Mean differences	
Length of postoperative hospital stay	5.79 [3.71-7.46]	<0.001
Hospitalization costs (1000 JPY)	147.96 [90.77-205.16]	<0.001

Abbreviations: IPTW, Inverse probability of treatment weighting; CI, confidence interval; JPY, Japanese yen

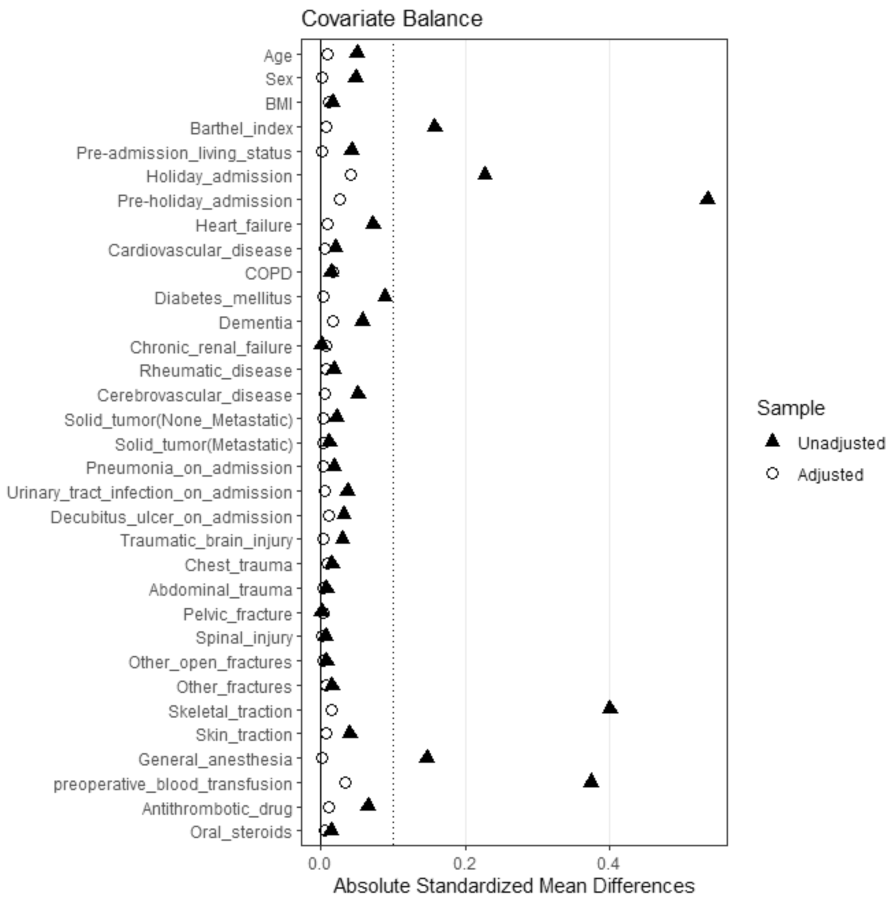

Supplemental Fig. 3 Covariate balance before and after IPTW adjustment in the cohort defined by early surgery within three days of admission

Abbreviations: IPTW, Inverse probability of treatment weighting; BMI, body mass index; COPD, chronic obstructive pulmonary disease

Supplemental Table 5 Baseline characteristics of the Early Surgery and Delayed Surgery groups, before and after IPTW adjustment in the cohort defined by early surgery within three days of admission

	Before IPTW			After IPTW		
	Early surgery group	Delayed surgery group	SMD	Early surgery group	Delayed surgery group	SMD
n	5,423 (48.9)	5,664 (51.1)	-	11,111.5	11,072.3	
Age, years, mean (SD)	81.91 (8.22)	82.31 (7.97)	0.050	82.24 (8.21)	82.18 (8.00)	0.008
Female, n (%)	4,719 (87.0)	5,017 (88.6)	0.048	9,751.7 (87.8)	9,715.2 (87.7)	0.001
BMI, kg/m^2, mean (SD)	22.23 (3.93)	22.29 (4.02)	0.015	22.19 (3.94)	22.23 (4.00)	0.011
Barthel Index (BI) at admission, mean (SD)	16.88 (28.60)	15.30 (26.74)	0.057	15.85 (27.52)	16.04 (27.28)	0.007

Pre-admission living status: Facility, n (%)	793 (14.6)	915 (16.2)	0.042	1,712.8 (15.4)	1,706.0 (15.4)	<0.001
Holiday admission, n (%)	1,385 (25.5)	2,037 (36.0)	0.227	3,678.1 (33.1)	3,457.5 (31.2)	0.041
Pre-holiday admission, n (%)	380 (7.0)	1,490 (26.3)	0.536	1759.3 (15.8)	1852.9 (16.7)	0.025
Heart failure, n (%)	320 (5.9)	437 (7.7)	0.072	805.2 (7.2)	776.4 (7.0)	0.009
Cardiovascular disease, n (%)	389 (7.2)	436 (7.7)	0.020	862.7 (7.8)	843.6 (7.6)	0.006
COPD, n (%)	44 (0.8)	53 (0.9)	0.013	114.0 (1.0)	97.9 (0.9)	0.015
Diabetes mellitus, n (%)	858 (15.8)	1,086 (19.2)	0.088	1,976.5 (17.8)	1,956.1 (17.7)	0.003
Dementia, n (%)	653 (12.0)	791 (14.0)	0.057	1,512.9 (13.6)	1,449.4 (13.1)	0.016
Chronic renal failure, n (%)	25 (0.5)	26 (0.5)	<0.001	51.7 (0.5)	56.3 (0.5)	0.006
Rheumatic disease, n (%)	199 (3.7)	227 (4.0)	0.018	418.3 (3.8)	432.9 (3.9)	0.008
Cerebrovascular disease, n (%)	380 (7.0)	474 (8.4)	0.051	865.7 (7.8)	849.8 (7.7)	0.004
Non-metastatic solid tumor, n (%)	280 (5.2)	320 (5.6)	0.022	602.2 (5.4)	594.9 (5.4)	0.002
Metastatic solid tumor, n (%)	99 (1.8)	95 (1.7)	0.011	198.6 (1.8)	194.3 (1.8)	0.003
Pneumonia at admission, n (%)	22 (0.4)	30 (0.5)	0.018	48.6 (0.4)	50.6 (0.5)	0.003
Urinary tract infection at admission, n (%)	22 (0.4)	38 (0.7)	0.036	52.4 (0.5)	56.6 (0.5)	0.005
Decubitus ulcer at admission, n (%)	30 (0.6)	46 (0.8)	0.031	86.4 (0.8)	77.0 (0.7)	0.001
Traumatic brain injury, n (%)	15 (0.3)	8 (0.1)	0.030	20.8 (0.2)	19.0 (0.2)	0.004
Chest injury, n (%)	25 (0.5)	32 (0.6)	0.015	67.1 (0.6)	60.6 (0.5)	0.008
Abdominal injury, n (%)	2 (0.0)	3 (0.1)	0.008	4.4 (0.0)	4.9 (0.0)	0.002
Pelvic injury, n (%)	18 (0.3)	19 (0.3)	0.001	32.9 (0.3)	34.3 (0.3)	0.002
Spinal injury, n (%)	39 (0.7)	38 (0.7)	0.006	80.8 (0.7)	79.0 (0.7)	0.002
Other open fractures, n (%)	6 (0.1)	5 (0.1)	0.007	10.1 (0.1)	9.2 (0.1)	0.002
Other fractures, n (%)	328 (6.0)	323 (5.7)	0.015	653.1 (5.9)	634.5 (5.7)	0.006

Skeletal traction, n (%)	1,910 (35.2)	3,103 (54.8)	0.401	4,952.5 (44.6)	5,007.7 (45.2)	0.013
Skin traction, n (%)	558 (10.3)	653 (11.5)	0.040	1,213.2 (10.9)	1,189.0 (10.7)	0.006
General anesthesia, n (%)	3,558 (65.6)	4,099 (72.4)	0.147	7,710.4 (69.4)	7,689.5 (69.4)	0.001
Preoperative blood transfusion, n (%)	586 (10.8)	1,411 (24.9)	0.375	2,172.6 (19.6)	2,023.6 (18.3)	0.034
Antithrombotic drug, n (%)	2,138 (39.4)	2,414 (42.6)	0.065	4,618.1 (41.6)	4,544.0 (41.0)	0.011
Oral steroids, n (%)	311 (5.7)	343 (6.1)	0.014	657.4 (5.9)	666.8 (6.0)	0.004

Abbreviations: IPTW, Inverse probability of treatment weighting; SMD, Standard mean difference; SD, Standard deviation; BMI, body mass index; COPD, chronic obstructive pulmonary disease

n in "After IPTW" columns represents the effective sample size after weighting

Supplemental Table 6 IPTW-adjusted odds ratios and mean differences of the clinical outcomes in the cohort defined by early surgery within three days of admission

Outcome	IPTW analysis	
	Estimate [95% CI]	P-value
Categorical variables	Odds ratio	
In-hospital mortality	0.84 [0.63-1.13]	0.249
Deep vein thrombosis	1.05 [0.83-1.33]	0.705
Pulmonary embolism	1.10 [0.60-2.03]	0.751
Pneumonia	0.88 [0.64-1.21]	0.436
Urinary tract infection	0.66 [0.52-0.84]	0.001
Delirium	1.37 [0.98-1.91]	0.063
Decubitus ulcer	0.79 [0.49-1.28]	0.341
Peroneal nerve palsy	1.53 [0.40-5.86]	0.538
Continuous variables	Mean differences	
Length of postoperative hospital stay	6.20 [4.56-7.85]	<0.001
Hospitalization costs (1000 JPY)	198.34 [147.78-248.90]	<0.001

Abbreviations: IPTW, Inverse probability of treatment weighting; CI, confidence interval; JPY, Japanese yen

Supplemental Table7 Odds ratios and mean differences of the clinical outcomes in the sensitivity analysis using a multivariate regression model

Outcome	Estimate [95% CI]	P-value
Categorical variables	Odds ratio	
In-hospital mortality	1.00 [0.72-1.38]	0.978
Deep vein thrombosis	0.96 [0.78-1.19]	0.717
Pulmonary embolism	0.87 [0.45-1.68]	0.672
Pneumonia	0.72 [0.51-1.01]	0.060
Urinary tract infection	0.72 [0.54-0.95]	0.020
Delirium	1.17 [0.81-1.70]	0.411
Decubitus ulcer	0.61 [0.40-0.94]	0.026
Peroneal nerve palsy	0.42 [0.11-1.64]	0.214
Continuous variables	Mean differences	
Length of postoperative hospital stay	5.72 [4.43-7.01]	<0.001
Hospitalization costs (1000 JPY)	163.92 [124.06-203.78]	<0.001

Abbreviations: CI, confidence interval; JPY, Japanese yen
